# ESG performance, auditing quality, and investment efficiency: Empirical evidence from China

**DOI:** 10.3389/fpsyg.2022.948674

**Published:** 2022-10-12

**Authors:** Wenbing Wang, Yanyan Yu, Xuan Li

**Affiliations:** ^1^School of Accountancy, Anhui University of Finance and Economics, Bengbu, Anhui, China; ^2^School of Accountancy, Shandong Technology and Business University, Yantai, Shandong, China

**Keywords:** ESG performance, auditing quality, investment efficiency, emerging countries, China

## Abstract

Dramatic changes in the business environment have created demands for additional information such as management discussions, governance information, and financial statement notes that go beyond the coverage of traditional financial reporting. Environmental, social, and governance (ESG) information can help gain stakeholder trust, reduce transaction costs, and improve investment efficiency. Taking Chinese A-share listed companies from 2011 to 2020 as a sample, we run fixed effect regressions to test the effect of ESG performance on investment efficiency. ESG performance is measured with the ESG score from the Bloomberg database. The results show that (a) good ESG performance significantly improves investment efficiency, (b) auditing quality partially mediates the relationship between ESG performance and investment efficiency, and (c) the role of ESG performance is stronger in non-state-owned enterprises, undeveloped regions, and firms with low accounting information quality. This paper contributes to the literature on ESG performance and provides references for ESG practice and sustainable corporate development in emerging countries.

## Introduction

ESG is the acronym for environmental, social, and governance. ESG information is an important part of nonfinancial information disclosure and complements traditional financial disclosure. ESG disclosure is valued because it helps stakeholders make better quality decisions (Zhang et al., 2020). Investors from developed countries have a higher level of ESG recognition. With the increasing foreign investment in China, foreign investors are driving Chinese companies to place greater emphasis on ESG practice and reporting (Cheng et al., 2014). In 2016, China’s seven ministries and commissions issued “*Guidelines for Establishing the Green Financial System”* to encourage green investments, curb pollution investments, build a green financial system, accelerate the green economy transformation, and promote efficient economic growth. In 2019, the Assets Management Association of China issued “the *Research Report on ESG Evaluation System of Listed Companies in China”* to highlight the concept of green development, emphasize the protection of the balance of interests among stakeholders, and promote modern corporate governance. In December 2020, “*the White Paper on ESG Development in China”* was issued to promote responsible investment and improve the ESG evaluation system of Chinese firms. Although the concept of ESG has attracted much attention, most scholars are committed to the research on ESG performance and enterprise value (Malik, 2015; Qureshi et al., 2019), and there is no relatively unified conclusion on the relationship between ESG performance and investment efficiency. Therefore, research on the effect of ESG performance on investment efficiency can both contribute to ESG literature and provide theoretical references for firms in China to improve investment efficiency.

The research theme of this paper is that good ESG performance will improve investment efficiency, and audit quality partially mediates the relationship between ESG performance and investment efficiency. First, good ESG performance may ease financing constraints and be more conducive to the external financing of enterprises (Lambert et al., 2007; Liu et al., 2021); Second, good ESG performance may reduce agency costs and effectively constrain management behavior (Lee and Kim, 2020). Third, social responsibility activities can integrate stakeholders into enterprise investment decisions and alleviate information asymmetry (Cui et al., 2018), thereby improving investment efficiency (Samet and Jarboui, 2017). In addition, in hypothesis 2, this paper discusses the impact of audit quality on investment efficiency, which paves the way for confirming the intermediary role of audit quality in the relationship between ESG performance and investment efficiency. The study found that high audit quality can reduce the quality of corporate earnings, improve the credibility of accounting information, and thus improve the efficiency of enterprise investment (Cheong and Zurbruegg, 2016; Palazuelos et al., 2018). Enterprises with good ESG performance tend to choose high-quality auditing to send positive signals to the outside world and reduce enterprise information asymmetry (Kim and Song, 2011). High audit quality can improve investment efficiency, which further confirms the intermediary role of audit quality in the relationship between ESG performance and investment efficiency.

There are three reasons why this paper chooses Chinese A-share listed companies as the research sample: first, emerging countries are relatively late in taking on ESG. Taking the Chinese market as the research sample enriches the research of ESG theory in emerging countries. Second, the organizational structure of Chinese enterprises is mostly pyramidal, which is complex and prone to agency problems, which makes us more interested in the relationship between ESG performance and the investment efficiency of Chinese enterprises (Hai et al., 2022). Third, the rapid development of the social economy makes the market competition increasingly intense, which greatly increases the complexity of the business environment of Chinese listed companies. The sustainable development of Chinese listed companies has become an important topic of academic research, which has led us to pay more attention to the research of Chinese listed companies (Xu and Bai, 2019).

We run regressions using a sample of Chinese A-share listed companies from 2011 to 2020. ESG performance is measured by the ESG score from the Bloomberg database. Our results show that good ESG performance can improve enterprise investment efficiency and that auditing quality mediates the relationship between ESG performance and investment efficiency. Further research shows that the positive relationship between ESG performance and investment efficiency is moderated by the nature of property rights, institutional environment, and accounting information quality. For non-state-owned enterprises (non-SOEs), firms in less-developed regions, and firms with low accounting information quality, ESG performance plays a stronger role in promoting investment efficiency. The results indicate that ESG practices promote Chinese firms’ sustainable development.

This paper makes the following contributions to the extant literature. First, most extant studies focus on a single ESG dimension, such as the environment, social responsibility, and corporate governance, and few studies take the three dimensions as a unit. This paper integrates the environmental, social, and governance dimensions into the same analytical framework to investigate the impact of Chinese firms’ ESG performance on investment efficiency, highlighting the overall effect of ESG in improving investment efficiency. Second, most extant ESG literature focuses on firm value/financial performance rather than investment efficiency. This paper contributes to the extant literature by highlighting the relationship between ESG and investment efficiency. Third, this paper enriches the extant ESG literature by investigating the mediating role of auditing quality in the relationship between ESG performance and investment efficiency.

The rest of this paper is organized as follows. Section 2 states the theoretical analysis and research assumptions and mainly analyzes the relationship between ESG performance, audit quality, and investment efficiency. Section 3 describes the variables and data used in this study and establishes a regression model. Section 4 is an empirical test and reports the regression results. Section 5 analyzes the heterogeneity. Section 6 mainly discusses the contribution of this study, the limitations and future directions.

## Theoretical analysis and research hypothesis

### ESG performance and investment efficiency

According to stakeholder theory, ESG practices help increase stakeholder trust, obtain stakeholder support, obtain strategic resources for corporate development, and improve investment efficiency (Liu et al., 2021). ESG performance improves investment efficiency in the following three ways. First, ESG practice reduces agency costs. Good ESG performance indicates that firms have a well-established corporate governance mechanism that can effectively constrain managers and mitigate agency problems (Lee and Kim, 2020). Positive ESG information reduces the negative influence of media coverage, buffers external pressures, reduces agency costs, and improves investment efficiency (Matten and Moon, 2008). ESG investments reduce corporate free cash flow and curb managerial short-sightedness, thereby mitigating agency costs and improving corporate investment efficiency (Samet and Jarboui, 2017).

Second, ESG performance can improve investment efficiency by mitigating financing constraints. ESG disclosure transmits nonfinancial information to investors and facilitates external financing (El Ghoul et al., 2011). ESG disclosure also increases external supervision and attention, helps uninformed investors obtain more information, and reduces the synchronization of stock prices (Kim et al., 2012). In addition, social responsibility performance is directly related to the approval of corporate refinancing in heavily polluting industries (Goss and Roberts, 2011).

Finally, ESG disclosure sends a positive signal to the market. Firms usually spend a certain amount to transmit nonfinancial information to society, which can reduce information asymmetry and facilitate investors in identifying quality companies (Spence, 1973). Good ESG performance reduces the information asymmetry between firms and investors and provides more information for stakeholders to make decisions, thereby decreasing the decision-making risk for investors and improving investment efficiency (Lins et al., 2017). Consequently, we propose the following hypothesis.

*H1*: ESG performance is positively associated with investment efficiency.

### Auditing quality and investment efficiency

Rapid economic growth and improving capital markets do not mean high investment efficiency. Chinese listed companies face serious problems of inefficient investment, including overinvestment and underinvestment (Qin and Song, 2009; Chen S. et al., 2011). According to the principal-agent theory, too much power of managers may breed short-sighted behavior, lead to the neglect of the long-term interests of the enterprise, generate excessive investment, cause uneven and unreasonable distribution of resources, and finally cause waste of enterprise resources and increase the risk of enterprise operation (Li, 2009; Chen et al., 2017). In contrast, if the management power is too constrained by other factors, it is easy to produce conservative investment behavior, resulting in insufficient investment. Insufficient investment can contribute to idle resources, increase the opportunity cost of enterprises, and damages the rights and interests of stakeholders (Stulz, 1990; Bertrand and Mullainathan, 2003). Improving investment efficiency has become an urgent problem to be solved.

Low investment efficiency is generally due to information asymmetry and principal-agent problems. High-quality accounting information can improve information transparency (Biddle and Hilary, 2006; Biddle et al., 2009). Auditing provides a guarantee for the quality of accounting information and has important reference value for stakeholders. Auditing quality affects investment efficiency in the following three ways. First, auditing can effectively alleviate information asymmetry, reduce investors’ decision-making risk, and improve investment efficiency through the signal transmission mechanism (Copley and Douthett, 2002). Second, high auditing quality reduces financing costs (Mansi et al., 2004; Lambert et al., 2007). High auditing quality enhances investors’ trust in financial information, alleviates information asymmetry, avoids insufficient investment caused by high financing costs, and thus improves investment efficiency (Bushman and Smith, 2001; Biddle et al., 2009). Third, auditing has the function of insurance and supervision (Chen H. et al., 2011). High auditing quality restricts the behavior of managers, improves the efficiency of corporate resource allocation, and inhibits inefficient investment (Bushman and Smith, 2001). High-quality auditing restrains the insufficient investment of firms and effectively solves investment problems (Copley and Douthett, 2002). The impact of auditing quality on investment efficiency varies with the nature of equity (Khurana and Raman, 2004; Chen H. et al., 2011). Consequently, we propose the following hypothesis.

*H2*: High auditing quality can improve the investment efficiency of firms.

### The mediating effect of auditing quality in the relationship between ESG performance and investment efficiency

By disclosing ESG information, firms transmit nonfinancial information to the outsiders, reduce information asymmetry, and improve investment efficiency (Lins et al., 2017). It is important to take effective measures to increase stakeholders’ trust in corporate disclosures. High-quality disclosure can improve corporate information transparency, reduce corporate information asymmetry, and alleviate agency problems (Mitton, 2002; Elaoud and Jarboui, 2017). External auditing is a guarantee of accounting information quality. The independence and objectivity of auditing can provide a guarantee for the quality of ESG disclosure. Auditing supervision has played a positive role in the development of ESG (Iatridis, 2011). Auditing standards require certified public accountants (CPAs) to check both the financial and nonfinancial information and measure firms’ sustainable operation ability more accurately. As important nonfinancial information, corporate social responsibility (CSR) reports are bound to be included in the auditing and constitute an important reference content for auditing risk assessment (Kolk and Margineantu, 2009).

As intermediaries, accounting firms use auditing standards to certify the ESG reports. The process ensures the authenticity and reliability of the information disclosed by companies, thus promoting the overall consistency between ESG information and financial information. The quality of accounting information quality audited by high-quality accounting firms is generally higher than that of small accounting firms (Becker et al., 1998; Francis and Yu, 2009). Therefore, to send a positive signal to the outside world, firms prefer to choose high-quality auditing, which further alleviates firms’ financing constraints, reduces their agency cost, and improves their investment efficiency (Jones and Raghunandan, 1998; Francis and Wang, 2008). High-quality auditing improves the efficiency of investor confidence by identifying accounting quality and thus improving the efficiency of capital allocation (Elaoud and Jarboui, 2017). Firms with good ESG performance are motivated to choose high-quality accounting firms to ensure the authenticity of accounting information and increase the trust of stakeholders in ESG information (Fan and Wong, 2005). Consequently, auditing quality plays a positive role in promoting investment efficiency (Bushman and Smith, 2001), and we propose the following hypothesis.

*H3*: Auditing quality plays a mediating role in the relationship between ESG performance and investment efficiency.

## Research design

### Data and sample

This paper uses China’s A-share listed companies from 2011 to 2020 as the research sample. The data are updated to 2020, which makes the research for this paper timely. The research period is the 10 years from 2011 to 2020, which makes the conclusions of the research persuasive. ESG performance data are from the Bloomberg database, other financial data are from the CSMAR and Wind databases, and the marketization degree data are from the “*Report on China’s Marketization Index by Province (2018).”* The data are processed as follows. First, due to the particularity of the financial statement calculation of the financial industry, we have excluded the samples from the financial industry and the real estate industry. Second, we exclude samples such as ST or ^*^ST to reduce the impact of outliers on the empirical results. Third, we exclude the samples with missing values. Our final full sample consists of 915 companies, corresponding to 7,933 firm-year observations. The data structure in this paper is an unbalanced panel (pooled cross-sectional and time series data). All continuous variables are winsorized at the 1 and 99% levels to reduce the influence of outliers.

### Variables

#### Dependent variable

Following Biddle et al. (2009), We use the residual obtained from model (1) to measure the efficient investment level of firms. the model is as follows:


(1) $$Inv_{i,t}=\partial_{0}+\partial_{1}{\mathrm{Salesgrowth}}_{i,t-1}+v_{i,t}$$

*Inv* represents the investment efficiency of firm *i* in year *t*. The investment level in year *t* = (cash paid for the purchase and construction of fixed assets, intangible assets, and other long-term assets + cash paid for the acquisition of subsidiaries and other business units + cash paid for investment–net cash recovered from the disposal of fixed assets, intangible assets and other long-term assets–net cash received from the disposal of subsidiaries and other business units–cash received from the recovery of investment)/total assets at the beginning of the period. *Salesgrowth* is the growth rate of operating revenue. $v_{i,t}$ is the regression residual of the Model (1). Through the industry and annual estimation model (1), the fitting value of the optimal investment level of the enterprise and the residual deviation from the optimal investment level of the enterprise are obtained. The residual part represents the deviation from the optimal investment level of the enterprise. The larger the absolute value of the residual is, the lower the investment efficiency.

#### Independent variable

Following Minutolo et al. (2019), we use the ESG score of the Bloomberg database to measure ESG performance. The Bloomberg database publicly publishes the level of CSR reporting to investors, including the ESG comprehensive index score and the E, S, and G single index scores. The score is based on the quality of ESG disclosure. The scoring range is 0–100. The more information a firm discloses, the higher the ESG score. Investors can access each company’s ESG score, scoring methodology, and score report data. The reasons for selecting the Bloomberg database are as follows. First, Bloomberg’s ESG data are obtained from company CSR/sustainability reports or other public sources, and the scores are more objective. Second, compared to other ESG ratings, Bloomberg’s ESG data cover a wider range and are more convincing.

#### Mediating variables

Auditing has a supervisory function, and external auditing is an assurance of accounting information quality. Following Teoh and Wong (1993), Hackenbrack and Hogan (2002), and Balsam et al. (2003), the size of an accounting firm determines auditing quality. In this paper, *Big4* is selected to measure auditing quality. *Big4* equals 1 if a firm selects the top four international accounting firms for auditing, and 0 otherwise.

#### Control variables

Following Bates (2005), we control firm size (Size), financial leverage (Lev), asset tangibility (Tang), financial performance (ROA), firm age (Age), market value to book ratio (MB), cash level (Cash), property right nature (SOE), free cash flow of enterprise (FCF), proportion of fixed assets (PPE), institutional environment (MKT), board size (Board) and industry and year dummy variables. The variables are defined as follows in Table 1.

**Table 1:** Definitions of variables.

Category	Variable name	Sign	Implication
Dependent variable	Investment efficiency	*Inv*	The absolute value of investment efficiency residual is calculated by the Biddle model.
Independent variable	ESG performance	*ESG*	ESG performance score calculated by Bloomberg database/100.
Mediating variable	Auditing quality	*Big4*	*Big4* equals 1 if a firm chooses the top four international accounting firms for auditing, and 0 otherwise.
Control variables	Firm size	*Size*	Natural logarithm of total assets of the company.
	Financial leverage	*Lev*	The ratio of total liabilities to total assets.
	Asset Tangibility	*Tang*	(Total assets–net intangible assets)/total assets.
	Profit level	*ROA*	The ratio of net profit to total assets.
	Firm age	*Age*	Natural logarithm of a firm’s establishment years.
	Ownership property	*SOE*	*SOE* equals 1 if a firm is a state-owned enterprise, and 0 otherwise.
	Market to book ratio	*MB*	The ratio of a firm’s market value to its book value.
	Cash level	*Cash*	The ratio of cash at bank and on hand to total assets.
	Free cash flow of enterprise	FCF	Free cash flow/total assets
	Proportion of fixed assets	PPE	Fixed assets/total assets
	Institutional environment	MKT	Market index by Region
	Board size	Board	Natural logarithm of the number of directors

### Model construction

We build fixed effect models (2) and (3) to test H1 and H2, respectively; *Industry FE* represents industry fixed effects; *Year FE* represents year fixed effects; *Province FE* represents Province fixed effects.


(2) $$\begin{array}{l} Inv_{i,t}=\beta_{0}+\beta_{1}ESG_{i,t}+\beta_{2}Size_{i,t}+\beta_{3}Lev_{i,t}\\ +\beta_{4}Tang_{i,t}+\beta_{5}ROA_{i,t}+\beta_{6}Age_{i,t}+\beta_{7}SOE_{i,t}\\ +\beta_{8}MB_{i,t}+\beta_{9}Cash_{i,t}+\beta_{10}FCF_{i,t}+\beta_{11}PPE_{i,t}\\ +\beta_{12}MKT_{i,t}+\beta_{13}Board_{i,t}+\beta_{14}Industry\;FE\\ +\beta_{15}Year\;FE+\beta_{16}Province\;FE+\varepsilon_{i,t} \end{array}$$

(3) $$\begin{array}{l} Inv_{i,t}=\beta_{0}+\beta_{1}Big\;4_{i,t}+\beta_{2}Size_{i,t}+\beta_{3}Lev_{i,t}+\beta_{4}Tang_{i,t}\\ +\beta_{5}ROA_{i,t}+\beta_{6}Age_{i,t}+\beta_{7}SOE_{i,t}+\beta_{8}MB_{i,t}+\beta_{9}Cash_{i,t}\\ +\beta_{10}FCF_{i,t}+\beta_{11}PPE_{i,t}+\beta_{12}MKT_{i,t}+\beta_{13}Board_{i,t}\\ +\beta_{14}Industry\;FE+\beta_{15}Year\;FE+\beta_{16}Province\;FE+\varepsilon_{i,t} \end{array}$$

Referring to the mediation effect test procedure proposed by Baron and Kenny (1986), we build fixed effect models (4) and (5) to test H3.


(4) $$\begin{array}{l} Big\;4_{i,t}=\beta_{0}+\beta_{1}ESG_{i,t}+\beta_{2}Size_{i,t}+\beta_{3}Lev_{i,t}+\beta_{4}Tang_{i,t}\\ +\beta_{5}ROA_{i,t}+\beta_{6}Age_{i,t}+\beta_{7}SOE_{i,t}+\beta_{8}MB_{i,t}+\beta_{9}Cash_{i,t}\\ +\beta_{10}FCF_{i,t}+\beta_{11}PPE_{i,t}+\beta_{12}MKT_{i,t}+\beta_{13}Board_{i,t}\\ +\beta_{14}Industry\;FE+\beta_{15}Year\;FE+\beta_{16}Province\;FE+\varepsilon_{i,t} \end{array}$$

(5) $$\begin{array}{l} Inv_{i,t}=\beta_{0}+\beta_{1}ESG_{i,t}+\beta_{2}Big\;4_{i,t}+\beta_{3}Size_{i,t}\\ +\beta_{4}Lev_{i,t}+\beta_{5}Tang_{i,t}+\beta_{6}ROA_{i,t}+\beta_{7}Age_{i,t}\\ +\beta_{8}SOE_{i,t}+\beta_{9}MB_{i,t}+\beta_{10}Cash_{i,t}+\beta_{11}FCF_{i,t}\\ +\beta_{12}PPE_{i,t}+\beta_{13}MKT_{i,t}+\beta_{14}Board_{i,t}\\ +\beta_{15}Industry\;FE+\beta_{16}Year\;FE+\beta_{17}Province\;FE+\varepsilon_{i,t} \end{array}$$

We conducted all our analyses in Stata 16.

## Results

### Descriptive statistics

Table 2 shows the descriptive statistical results of the main variables. The average investment efficiency of the sample firms is 0.0389 indicating that the investment efficiency of China’s A-share listed companies is generally low. The minimum value of investment efficiency is 0.0006 and the maximum value is 0.2282, indicating that the investment efficiency of different firms varies greatly. The average ESG performance is 0.2090, the minimum value is 0.0909, and the maximum value is 0.4421, indicating that there is a wide variation in the ESG performance of Chinese firms. The mean value of auditing quality is 0.1083 and the standard deviation is 0.3108, indicating that the importance attached to audit quality varies widely among firms. The average tangible value of assets is 0.9482, the lowest is 0.6841, and the highest is 1. It shows that most of the enterprises are tangible assets and less intangible assets. The average ROA of the company’s profitability is 0.0433, the minimum value is −0.2934, and the maximum value is 0.2027, indicating that the overall profitability of the enterprise is low, and the profitability gap between different enterprises is large. The descriptive statistical results of other variables such as Lev and SOE are similar to the conclusions of existing research (Hai et al., 2022), so they will not be repeated.

**Table 2:** Descriptive statistics.

Variables	Sample	Mean	*SD*	Min.	Max.
*Inv*	7,933	0.0389	0.0388	0.0006	0.2282
*ESG*	7,933	0.2090	0.0677	0.0909	0.4421
*Big4*	7,933	0.1083	0.3108	0.0000	1.0000
*Size*	7,933	22.9725	1.1030	19.6770	24.6748
*Lev*	7,933	0.4610	0.1914	0.0508	0.9306
*Tang*	7,933	0.9482	0.0565	0.6841	1.0000
*ROA*	7,933	0.0433	0.0605	−0.2934	0.2027
*Age*	7,933	2.8442	0.3395	0.6931	3.6889
*SOE*	7,933	0.5396	0.4985	0.0000	1.0000
*MB*	7,933	1.9373	1.2693	0.8614	8.7438
*Cash*	7,933	0.1645	0.1140	0.0151	0.6624
*FCF*	7,933	0.0133	0.0960	−0.4464	0.2562
*PPE*	7,933	0.2546	0.1811	0.0000	0.9542
*MKT*	7,933	8.2064	1.9495	3.3700	11.3100
*Board*	7,933	2.1863	0.2021	1.6094	2.7081

It can be seen from Table 3 that industry and public utilities account for the highest proportion, with 6,013 observed values in the industry, accounting for 75.80%. There are 1,135 observed values in public utilities, accounting for 14.31%. It can be seen from Table 4 that the overall investment level of the four industries is 0.0389. The average ESG of the four industries is 0.2090, of which the industrial ESG performs well, with an average of 0.2122, and the comprehensive ESG performs poorly, with an average of 0.1825.

**Table 3:** Sample distribution across sectors.

Industry code	Industry	*N*	%
0002	Public utility	1,135	14.31%
0004	Comprehensive	417	5.26%
0005	Industrial	6,013	75.80%
0006	Business	368	4.63%
	Total	7,933	100.00%

**Table 4:** Descriptive statistics (Mean).

Variables	Public utility	Comprehensive	Industrial	Business	Total
*Inv*	0.0383	0.0336	0.0399	0.0300	0.0389
*ESG*	0.2046	0.1825	0.2122	0.1988	0.2090
*Big4*	0.1621	0.0024	0.1026	0.1549	0.1083
*Size*	23.0903	22.8411	22.9428	23.2427	22.9725
*Lev*	0.4157	0.5007	0.4611	0.5541	0.4610
*Tang*	0.9288	0.9452	0.9517	0.9532	0.9482
*ROA*	0.0460	0.0298	0.0442	0.0355	0.0433
*Age*	2.7627	2.9601	2.8459	2.9358	2.8442
*SOE*	0.6555	0.4077	0.5237	0.5924	0.5396
*MB*	1.9997	2.0027	1.9473	1.5059	1.9373
*Cash*	0.1862	0.1527	0.1596	0.1909	0.1645
*FCF*	0.0138	0.0104	0.0136	0.0111	0.0133
*PPE*	0.2430	0.1777	0.2674	0.1684	0.2546
*MKT*	8.8504	7.8354	8.0679	8.9053	8.2064
*Board*	2.1892	2.1636	2.1863	2.2032	2.1863

### Correlation analysis

The correlation coefficient between the main variables is shown in Table 5. It can be seen from the table that the correlation coefficient between ESG performance (*ESG*) and investment efficiency (*Inv*) is negative and significant at the 1% level. It is preliminarily proven that ESG performance can restrict the behavior of management, reduce enterprise agency costs, and improve enterprise investment efficiency. The correlation coefficient between auditing quality (*Big4*) and investment efficiency (*Inv*) is significantly negative, indicating that auditing quality can alleviate the inefficient investment of firms. The higher the quality of accounting firms, the more rigorous the implementation of auditing, which can better give play to the function of external supervision and improve the investment efficiency of firms. In addition, the correlation coefficient between other variables is small, indicating that there is no serious multicollinearity problem between variables.

**Table 5:** Correlations.

	*Inv*	*ESG*	*Big4*	*Size*	*Lev*	*Tang*	*ROA*	*Age*	*State*	*MB*	*Cash*	*FCF*	*PPE*	*Board*
*Inv*	1													
*ESG*	−0.053^***^	1												
*Big4*	−0.027^***^	0.342^***^	1											
*Size*	−0.050^***^	0.394^***^	0.309^***^	1										
*Lev*	0.010	0.133^***^	0.086^***^	0.436^***^	1									
*Tang*	−0.041^***^	−0.056^***^	−0.057^***^	−0.015^**^	0.001	1								
*ROA*	0.039^***^	−0.0060	0.052^***^	0.056^***^	−0.339^***^	0.039^***^	1							
*Age*	−0.098^***^	0.204^***^	0.040^***^	0.224^***^	0.227^***^	−0.016^**^	−0.095^***^	1						
*State*	−0.029^***^	0.202^***^	0.135^***^	0.300^***^	0.259^***^	−0.053^***^	−0.051^***^	0.221^***^	1					
*MB*	0.020^***^	−0.160^***^	−0.092^***^	−0.443^***^	−0.207^***^	−0.001	0.085^***^	0.009	−0.134^***^	1				
*Cash*	−0.019**	−0.119^***^	−0.054^***^	−0.248^***^	−0.374^***^	0.135^***^	0.227^***^	−0.187^***^	−0.106^***^	0.133^***^	1			
*FCF*	−0.162^***^	0.054^***^	0.046^***^	0.082^***^	0.012^*^	−0.036^***^	0.221^***^	0.003	0.022^***^	−0.037^***^	−0.118^***^	1		
*PPE*	0.088^***^	0.151^***^	0.072^***^	0.166^***^	0.175^***^	−0.031^***^	−0.099^***^	0.045^***^	0.227^***^	−0.153^***^	−0.369^***^	0.087^***^	1	
*MKT*	−0.076^***^	0.116^***^	0.069^***^	0.056^***^	−0.094^***^	0.086^***^	0.018^***^	0.102^***^	−0.175^***^	0.012	−0.001	0.009	−0.178^***^	1
*Board*	−0.012	0.115^***^	0.099^***^	0.259^***^	0.139^***^	−0.056^***^	0.040^***^	0.033^***^	0.257^***^	−0.155^***^	−0.045^***^	0.034^***^	0.147^***^	−0.107^***^

^***^p < 0.01, ^**^p < 0.05, ^*^p < 0.1.

### Regression results

Model (2) tests the impact of ESG performance on investment efficiency. Column (1) of Table 6 shows that the coefficient of *ESG* is −0.0220 and significant at the 1% level. Consistent with H1, the results indicate that ESG performance can effectively improve investment efficiency. Model (3) tests the relationship between auditing quality and investment efficiency. Column (2) of Table 6 shows that the regression coefficient of *Big4* is −0.0054 and significant at the 1% level, indicating that higher-quality accounting firms improve investment efficiency. The regression results are consistent with H2.

**Table 6:** The effect of ESG performance on investment efficiency.

Variables	(1)	(2)	(3)	(4)
	*Inv*	*Inv*	*Big4*	*Inv*
*ESG*	−0.0220^***^		1.2103^***^	−0.0164^**^
	(−3.2289)		(17.7080)	(−2.3125)
*Big4*		−0.0054^***^		−0.0046^***^
		(−4.0075)		(−3.2381)
Controlled variable	Control	Control	Control	Control
Industry fixed effect	Yes	Yes	Yes	Yes
Year fixed effect	Yes	Yes	Yes	Yes
Provincial fixed effect	Yes	Yes	Yes	Yes
Sobel test				−0.0035^**^
*N*	7,933	7,933	7,933	7,933
Adj. *R*^2^	0.1116	0.1120	0.2310	0.1125

*Robust t-*statistics in parentheses; ^***^p < 0.01, ^**^p < 0.05, ^*^p < 0.1.

According to the stepwise method of Baron and Kenny (1986), if the independent variable *X* affects the dependent variable *Y* by influencing variable *M*, *M* is called an intermediary variable. In this paper, ESG performance is the independent variable *X*, investment efficiency is the dependent variable *Y*, and auditing quality is the mediating variable *M*. First, we test the significance of coefficient c of model (6). In the second step, we test the significance of coefficient *a* of model (7) and coefficient b of model (8). If both *a* and *b* are significant, there is an intermediary effect. The third step is to test whether there is a full mediating effect or a partial mediating effect. If *c*’ is significant, it is a partial mediating effect. If *c*’ is not significant, it is a complete mediating effect. Column (3) of Table 6 shows that the coefficient of *ESG* is 1.2103 and significant at the 1% level, indicating that firms with good ESG performance are more likely to choose high-quality accounting services to obtain stakeholders’ trust. In column (4) of Table 6, the coefficient of *ESG* is −0.0164 and significant at the 5% level, indicating that good ESG performance can improve enterprise investment efficiency. The coefficient of *Big4* is −0.0046 and significant at the 1% level, indicating that high auditing quality can improve investment efficiency. The coefficient of *ESG* increased from −0.0220 to −0.0164, representing an increase of 25.45%, and the model passed the Sobel test. Therefore, auditing quality plays a partial mediating role in the relationship between ESG performance and investment efficiency. Because the coefficient of *ESG* increases after adding the auditing quality variable *Big4* to Model (2), the mediating effect is positive. The regression results are consistent with H3.


(6) $$Y=cX+e_{1}$$

(7) $$M=aX+e_{2}$$

(8) $$Y=c^{\prime }X+bM+e_{3}$$

### Robustness tests

#### ESG performance and investment efficiency

First, we replace the independent variable to run regressions. In Model (2), we use the Huazheng ESG rating to measure ESG performance (*ESG2*). The higher the Huazheng ESG rating is, the better the ESG performance. The Huazheng ESG ratings CCC-AAA are assigned as 1–9 in order, i.e., ESG performance rating C is 1, CC is 2, CCC is 3, and so on. Table 7 column (1) shows the results of the regression with ESG2. The coefficient of *ESG2* is −0.0005 and significantly negative at the 5% level. The results show that ESG2 can also improve investment efficiency, which further proves the H1 hypothesis that ESG performance can improve investment efficiency, and that this result is robust.

**Table 7:** Robust tests.

Variables	(1)	(2)	(3)
	*Inv*	*Inv*	*Inv*1
*ESG*			−0.0310^***^
			(−3.4249)
*ESG2*	−0.0005^**^		
	(−2.0121)		
*ESG3*		−0.0015^**^	
		(−2.5608)	
Controlled variable	Control	Control	Control
Industry fixed effect	Yes	Yes	Yes
Year fixed effect	Yes	Yes	Yes
Provincial fixed effect	Yes	Yes	Yes
*N*	10,398	10,398	7,940
Adj. *R*^2^	0.0915	0.0918	0.0758

*Robust t-*statistics in parentheses; ^***^p < 0.01, ^**^p < 0.05, ^*^p < 0.1.

Second, this paper uses a more direct method to measure ESG performance (ESG3). We assign a Huazheng ESG rating CCC-AAA of 1–3 in order. The ESG performance rating C is 1, B is 2, and A is 3. Column (2) of Table 7 shows the regression results with *ESG3*. The coefficient of *ESG3* is −0.0015 and significantly negative at the 5% level, indicating that the regression results are robust. The results show that ESG3 can also improve investment efficiency, which shows that replacing independent variables will not change the conclusion of H1; that is, the conclusion of our H1 hypothesis is robust.

Third, we replace the dependent variable to run regressions. Following Richardson (2006), this paper measures the level of efficiency investment by the residual of Model (9). The greater the absolute value of the residual is, the lower the investment efficiency level.


(9) $$\begin{array}{l} Inv_{i,t}=\beta_{0}+\beta_{1}Growth_{i,t-1}+\beta_{2}Lev_{i,t-1}+\\ \beta_{3}Age_{i,t-1}+\beta_{4}Cash_{i,t-1}+\beta_{5}Size_{i,t-1}+\\ \beta_{6}Ret_{i,t-1}+\beta_{7}Inv_{i,t-1}+\sum \mathrm{Industry}+\sum \mathrm{Year}+v_{i,t} \end{array}$$

$Growth_{i,t-1}$ represents the growth rate of prime operating revenue, $Ret_{i,t-1}$ represents the annual return of stocks, and $Inv_{i,t-1}$ is the investment efficiency level of the previous year. $v_{i.t}$ is the regression residual, and its absolute value shows the level of investment efficiency. Column (3) of Table 7 reports the regression results. The coefficient of *ESG* is −0.0310 and significantly negative at the 1% level, indicating that good ESG performance can improve investment efficiency. The results show that changing the measurement method of dependent variables can also lead to the conclusion that good ESG performance can improve investment efficiency, which further proves the credibility of the conclusion of H1; that is, the conclusion of our H1 is robust.

#### Audit quality and investment efficiency

First, we run regression with a new independent variable: Audit1. In Model (3), we use audit opinion to measure audit quality (Audit1). An unqualified opinion is assigned a value of 1 and a nonunqualified opinion is assigned a value of 0. Table 8 column (1) shows the results of the regression with the Audit1. The coefficient of Audit1 is −0.0036 and significantly negative at the 5% level. The results still show that high audit quality can improve investment efficiency, that is, changing the measurement method of audit quality does not change the conclusion of hypothesis 2, indicating that the conclusion of H2 is robust.

**Table 8:** Robust tests.

Variables	(1)	(2)	(3)
	*Inv*	*Inv*	*Inv*1
*Big4*			−0.0023^**^
			(−2.0055)
*Audit1*	−0.0036^**^		
	(−2.0154)		
*Audit2*		−0.0031^***^	
		(−4.7525)	
Controlled variable	Control	Control	Control
Industry fixed effect	Yes	Yes	Yes
Year fixed effect	Yes	Yes	Yes
Provincial fixed effect	Yes	Yes	Yes
*N*	16,730	16,609	16,644
Adj. *R*^2^	0.0862	0.0902	0.0724

*Robust t-*statistics in parentheses; ^***^p < 0.01, ^**^p < 0.05, ^*^p < 0.1.

Second, this paper uses a more direct method to measure audit quality (Audit2) by using the natural logarithm of audit fees as a substitute variable for audit quality. The higher the audit cost is, the higher the audit quality. Table 8 column (2) shows the results of the regression with the Audit2. The coefficient of Audit2 is −0.0031 and significantly negative at the 1% level, indicating that good audit quality can improve investment efficiency. That is, changing the measurement method of audit quality does not change the conclusion of hypothesis 2, which shows that the conclusion of H2 is robust.

Third, following Richardson (2006), this paper measures the level of efficiency investment by the residual of Model (6). The greater the absolute value of the residual is, the lower the investment efficiency level. Column (3) of Table 8 reports the regression results. The coefficient of audit quality (*Big4*) is −0.0023 and significantly negative at the 5% level, indicating that good audit quality can improve investment efficiency. The results show that by changing the measurement method of dependent variables, the conclusion is still consistent with H2, which further proves the credibility of H2.

### Endogeneity

First, we add control variables to address the influence of the missing variables. For the unobservable missing variables that may exist in the model, which may affect the conclusion, we adopt adding control variables to solve the problem of endogeneity caused by missing variables. Given that the ownership concentration (*fhold*) and the proportion of independent directors (*indep*) will also affect investment efficiency, we add the two variables to Model (2). Column (1) of Table 9 shows the regression results. The coefficient of *ESG* is −0.0222 and significantly negative at the 1% level, which shows that the addition of control variables does not affect the conclusion of H1; that is, there is no influence of omitted variables on endogeneity, and our result is still robust.

**Table 9:** Endogeneity test with additional control variables.

Variables	(1)
	*Inv*
*ESG*	−0.0222^***^
	(−3.2268)
Controlled variable	Control
Industry fixed effect	Yes
Year fixed effect	Yes
Provincial fixed effect	Yes
*N*	7,933
Adj. *R*^2^	0.1121

*Robust t-*statistics in parentheses; ^***^p < 0.01, ^**^p < 0.05, ^*^p < 0.1.

Second, we run regressions to alleviate the influence of two-way causality. We lag the independent variables by one-, two-, and three- periods for regressions, respectively. The results are shown in Table 10. The coefficient of 1 year lagged *ESG* is −0.0243 and significant at the 1% level, the coefficient of two-years lagged *ESG* is −0.0247 and significant at the 1% level, and the coefficient of three-years lagged *ESG* is −0.0247 and significant at the 1% level. This shows that good ESG performance can improve investment efficiency; that is, there is no problem of endogeneity caused by two-way causality, and our conclusion is robust.

**Table 10:** Endogeneity test with hysteretic variables.

Variables	(1)	(2)	(3)
	*Inv*	*Inv*	*Inv*
			
*L.ESG*	−0.0243^***^		
	(−3.3493)		
*L2.ESG*		−0.0247^***^	
		(−3.3261)	
*L3.ESG*			−0.0247^***^
			(−3.0473)
Controlled variable	Control	Control	Control
Industry fixed effect	Yes	Yes	Yes
Year fixed effect	Yes	Yes	Yes
Provincial fixed effect	Yes	Yes	Yes
*N*	7,189	6,349	5,461
Adj. *R*^2^	0.0949	0.0822	0.0792

*Robust t-*statistics in parentheses; ^***^p < 0.01, ^**^p < 0.05, ^*^p < 0.1.

Third, we run the two-stage least squares (2SLS) regressions. Following Benlemlih and Bitar (2018), we use the average ESG score of other firms in the same province as an instrumental variable (IV1). Additionally, considering that the firm’s earliest ESG score has an impact on current ESG performance but is not related to the current model disturbance, we use the firm’s earliest ESG performance as the instrumental variable (IV2). We run the 2SLS regressions for endogenous tests. The results are shown in Table 11. In the first-stage regression, the coefficients of instrumental variables IV1 and IV2 are significantly positive, and the *F* value is far greater than 10, indicating that there is no problem with weak instrumental variables, and the tool variables we selected are valid. We also conduct an overidentification test on the instrumental variables. The *p* value of the Sargan test is 0.2225, which is greater than 0.1, rejecting the assumption that the instrumental variable is endogenous and indicating that the two instrumental variables are effective. In the second-stage regression, the *ESG* coefficient is −0.0602 and significant at the 1% level, which shows that good ESG performance can improve investment efficiency, indicating that our result is robust.

**Table 11:** Results of two-stage least squares regression.

Variables	Stage I	Stage II
	*ESG*	*Inv*
*ESG*		−0.0602^***^
		(−4.8017)
*IV1*	0.4007^***^	
	(11.4703)	
*IV2*	0.5463^***^	
	(49.4241)	
Controlled variable	Control	Control
Industry fixed effect	Yes	Yes
Year fixed effect	Yes	Yes
Provincial fixed effect	No	No
Sargan test value of *p*		0.2225
*N*	7,933	7,933
Adj. *R*^2^	0.4994	0.1050

*Robust t-*statistics in parentheses; ^***^p < 0.01, ^**^p < 0.05, ^*^p < 0.1.

## Additional analyses

### Heterogeneous impacts of ESG performance on the investment efficiency of SOEs and non-SOEs

First, SOEs and non-SOEs have different motivations for ESG disclosure. SOEs have a dual identity as both a political entity and a market entity. They consider national policies and social impacts first and economic returns second. Non-SOEs have only one identity as market participants, and the main purpose of their ESG disclosure is to obtain higher economic returns. Second, SOEs and non-SOEs have different ESG disclosure focuses. SOEs first respond to national policies and conduct ESG practices following national development directions. However, non-SOEs focus more on stakeholders’ needs to obtain more economic returns. As shown in columns (1) and (2) of Table 12, the coefficient of *ESG* of the non-SOE group is −0.0625 and significant at the 1% level, but the coefficient of *ESG* of the SOE group is not significant. The results show that ESG performance is positively associated with the investment efficiency of non-SOEs. However, the relationship does not exist in SOEs. Non-SOEs are mainly driven by market competition to engage in CSR activities and good ESG performance can improve investment efficiency and financial performance (Sun et al., 2019, 2020). In contrast, SOEs are mainly driven by institutional pressures to be socially responsible, so the ESG performance in SOEs does not significantly affect investment efficiency.

**Table 12:** Results of grouped regressions.

Variables	Non-SOEs	SOEs	Low marketization	High marketization	Low accounting information quality	High accounting information quality
	(1)	(2)	(3)	(4)	(5)	(6)
	*Inv*	*Inv*	*Inv*	*Inv*	*Inv*	*Inv*
*ESG*	−0.0625^***^	0.0040	−0.0489^***^	−0.0128	−0.0374^***^	−0.0158
	(−4.5057)	(0.4126)	(−3.7243)	(−1.2399)	(−2.9822)	(−1.4779)
Control variables	Control					
Industry fixed effect	Yes					
Year fixed effect	Yes					
Provincial fixed effect	Yes					
*N*	3,652	4,281	3,915	4,018	3,771	4,162
Adj. *R*^2^	0.1571	0.0888	0.1224	0.0978	0.1105	0.1083

*Robust t-*statistics in parentheses; ^***^p < 0.01, ^**^p < 0.05, ^*^p < 0.1.

### Grouped regressions by marketization

China’s economy has grown rapidly in recent years, but as a large emerging market country, it also suffers from serious economic development imbalances. In China’s more market-oriented regions, investment is less affected by economic instability and is more efficient and stable. Developed regions have more intense competition, higher market fairness, and better levels of information communication. The pressure on companies in developed regions is also relatively high. Good communication mechanisms in developed regions can strengthen external monitoring, and shareholders are more efficient in “voting with their feet.” Companies with a better external environment have more access to information, which improves the efficiency of resource allocation (Pinkowitz et al., 2006). The role of external governance is better in developed regions, and the role of ESG disclosure is weakened. In contrast, the marginal utility of ESG performance is greater in less developed regions, which can better alleviate the problems caused by information asymmetry and improve investment efficiency.

We define firms with less than the median marketization level of all A-share listed companies as low marketization and firms with greater than the median marketization of all A-share listed companies as high marketization. As shown in columns (3) and (4) of Table 12, the coefficient of *ESG* of the low marketization group is −0.0489 and significant at the 1% level, and the coefficient of *ESG* of the high marketization group is not significant. This is consistent with our expectations. The marginal effect of practicing ESG in underdeveloped regions is greater; that is, good ESG performance in underdeveloped regions can better improve investment efficiency. Developed regions have better external governance, and the relationship between ESG performance and investment efficiency is not obvious.

### Grouped regressions by accounting information quality

As mentioned above, ESG performance can alleviate information asymmetry and improve investment efficiency. However, ESG performance has a “ceiling effect” on the mitigation of information asymmetry; that is, when the information asymmetry between enterprises and stakeholders is effectively filled by other information, the impact of ESG information disclosure on investment efficiency is significantly reduced. Stakeholders in the capital market obtain important information about a company mainly from financial reports. Therefore, if the quality of accounting information of a company is relatively high, the marginal utility of ESG disclosure will be greatly reduced when the financial information can better meet the needs of stakeholders.

This paper argues that ESG performance can only have a significant impact on investment efficiency in firms with low accounting information quality. We refer to the Jones model modified by Dechow et al. (1995) to measure accounting information quality. The greater the absolute value of manipulation accrued profit, the lower the quality of accounting information. We take the median of the absolute value of all A-share listed companies’ manipulation of accrued profits as the standard to measure the quality of accounting information. Enterprises greater than the median are allocated to the low accounting information quality group, and enterprises less than the median absolute value are allocated to the high accounting information quality group. Columns (5) and (6) of Table 12 show the results of grouped regressions by accounting information quality. The coefficient of *ESG* of the group with low accounting information quality is −0.0374 and significant at the 1% level, and the coefficient of *ESG* of the group with high accounting information quality is not significant. The results show that good ESG performance can improve investment efficiency in samples with low accounting information quality, but this effect is not obvious in samples with high accounting information quality. This is consistent with our expectations. In areas with low-quality accounting information, ESG information disclosure can alleviate information asymmetry and improve investment efficiency. In areas with high-quality accounting information, the marginal utility of ESG information disclosure is greatly reduced.

## Conclusion

This paper empirically examines the impact of ESG performance on investment efficiency using a sample of Chinese A-share listed companies from 2011 to 2020. The results show that ESG performance can significantly improve investment efficiency. Audit quality plays a partial mediating role in the relationship between ESG performance and investment efficiency. Further tests show that the impacts of ESG performance on investment efficiency are influenced by the nature of property rights, institutional environment, and accounting information quality. The promotion effect of ESG performance on investment efficiency is stronger for non-SOEs, firms in less developed regions, and firms with low-quality accounting information.

This study makes several contributions to the current literature. First, this paper highlights the overall effect of ESG in improving investment efficiency. Most extant studies focus on the single ESG dimension, such as the environment, social responsibility, and corporate governance (Bostian et al., 2016; Chen et al., 2018; Castelló-Taliani et al., 2021). Few studies have taken these three dimensions as a unit. Integrating environmental, social, and governance dimensions into one analytical framework, this paper highlights the overall role of ESG in improving investment efficiency.

Second, this paper contributes to the extant literature by highlighting the relationship between ESG and investment efficiency. Most extant ESG literature focuses on company value and financial performance (Yoon et al., 2018; Taliento et al., 2019; Broadstock et al., 2020), but little attention is given to investment efficiency. ESG reports provide mainly nonfinancial information that makes up for traditional financial information disclosure deficiencies, which may improve the reasonability of investment decisions and help firms with sustainable development. Our empirical results provide a new understanding of ESG performance outcomes by highlighting the positive relationship between ESG performance and investment efficiency.

Third, this paper enriches the existing ESG literature by investigating the mediating role of auditing quality in the relationship between ESG performance and investment efficiency. We find that audit quality plays a partially mediating role in the relationship between ESG performance and investment efficiency. External audits can provide a certain degree of assurance on the quality of ESG information by their independence and objectivity. Accounting firms use auditing standards to verify the ESG reporting, ensuring the truthfulness and reliability of ESG information. Top accounting firms signal higher quality of ESG information of audited companies. Firms are more willing to choose high-quality audits to send positive signals to outsiders, which alleviates financing constraints, reduces agency costs, and improves investment efficiency (Zeng et al., 2019). With these results, this paper sheds new light on studies related to the outcomes of ESG performance.

There are some important changes that need to be made. At the firm level, firms should actively engage in ESG practices. First, firms should integrate ESG philosophy into their culture to build a good business image, enhance the trust of investors, and alleviate financing constraints. Second, firms should incorporate ESG practices into management systems and specific businesses, such as product development, employee training, social charity, etc. Through these measures, the degree of information asymmetry between firms and external investors can be reduced and corporate investment efficiency can be improved. At the market level, investors should incorporate ESG performance into investment decisions. First, while considering financial information, investors should also include nonfinancial information, such as environmental information, social responsibility information, and corporate governance information, in the decision-making framework to integrate ESG with investors’ strategic objectives, thus making more scientific decisions and reducing investment risks. Second, investors should play an external governance role to actively promote corporate ESG practices and sustainable development and improve the efficiency of capital allocation. At the institutional level, the government should play a regulatory role in promoting corporate ESG practices. First, the ESG information disclosure system in China is not perfect at present, and the quality of ESG information disclosure of many firms is poor. The government should work on improving the ESG information disclosure system and promote firms to continuously improve the ESG information quality. Second, the government should intervene less in the market and establish a good institutional environment for corporate ESG practices, thus improving the efficiency of market capital allocation. Finally, the government should advance the regulatory system for ESG disclosure and strengthen the penalties for false ESG information.

The generalizability of these results is subject to certain limitations. First, we examine the impact of ESG performance on investment efficiency only for Chinese listed firms but do not cover other emerging countries. The sample size should be expanded to include firms from all emerging countries in future studies. Second, this paper quantifies ESG performance with ESG reporting scores. The quality of ESG ratings may be undermined by imperfect and noncomparable ESG reporting rules.

## Data availability statement

The raw data supporting the conclusions of this article will be made available by the authors, without undue reservation.

## Author contributions

WW, YY, and XL completed the research design together. WW provided research assistance and support. YY and XL collected and analyzed the data and wrote the manuscript all up. All authors contributed to the article and approved the submitted version.

## Funding

This work is supported by the National Office for Philosophy and Social Sciences under grant number 21BGL097.

## Conflict of interest

The authors declare that the research was conducted in the absence of any commercial or financial relationships that could be construed as a potential conflict of interest.

## Publisher’s note

All claims expressed in this article are solely those of the authors and do not necessarily represent those of their affiliated organizations, or those of the publisher, the editors and the reviewers. Any product that may be evaluated in this article, or claim that may be made by its manufacturer, is not guaranteed or endorsed by the publisher.

## References

[ref1] Balsam S. Krishnan J. Yang J. S. (2003). Auditor industry specialization and earnings quality. Audit. J. Pract. Theory 22, 71–97. doi: 10.2308/aud.2003.22.2.71

[ref2] Baron R. M. Kenny D. A. (1986). The moderator-mediator variable distinction in social psychological research: conceptual, strategic, and statistical considerations. J. Pers. Soc. Psychol. 51, 1173–1182. doi: 10.1037/0022-3514.51.6.1173, PMID: 3806354

[ref3] Bates T. W. (2005). Asset sales, investment opportunities, and the use of proceeds. J. Finance 60, 105–135. doi: 10.1111/j.1540-6261.2005.00726.x

[ref4] Becker C. L. Defond M. L. Jiambalvo J. J. Subramanyam K. R. (1998). The effect of audit quality on earnings management. Contemp. Account. Res. 15, 1–24. doi: 10.1111/j.1911-3846.1998.tb00547.x

[ref5] Benlemlih M. Bitar M. (2018). Corporate social responsibility and investment efficiency. J. Bus. Ethics 148, 647–671. doi: 10.1007/s10551-016-3020-2

[ref6] Bertrand M. Mullainathan S. (2003). Enjoying the quiet life? Corporate governance and managerial preferences. J. Polit. Econ. 111, 1043–1075. doi: 10.1086/376950

[ref7] Biddle G. C. Hilary G. (2006). Accounting quality and firm-level capital investment. Account. Rev. 81, 963–982. doi: 10.2308/accr.2006.81.5.963

[ref8] Biddle G. C. Hilary G. Verdi R. S. (2009). How does financial reporting quality relate to investment efficiency? J. Acc. Econ. 48, 112–131. doi: 10.1016/j.jacceco.2009.09.001

[ref9] Bostian M. Fare R. Grosskopf S. Lundgren T. (2016). Environmental investment and firm performance: A network approach. Energy Econ. 57, 243–255. doi: 10.1016/j.eneco.2016.05.013

[ref10] Broadstock D. C. Chan K. Cheng L. Wang X. (2020). The role of ESG performance during times of financial crisis: evidence from COVID-19 in China. Finance Res. Lett. 38:101716. doi: 10.2139/ssrn.3627439 PMC7425769 32837385

[ref11] Bushman R. M. Smith A. J. (2001). Financial accounting information and corporate governance. J. Account. Econ. 32, 237–333. doi: 10.1016/S0165-4101(01)00027-1

[ref12] Castelló-Taliani E. Giralt Escobar S. da Rosa F. S. (2021). Environmental disclosure: study on efficiency and alignment with environmental priorities of Spanish ports. Sustainability 13:1791. doi: 10.3390/su13041791

[ref13] Chen H. Chen J. Z. Lobo G. J. Wang Y. (2011). Effects of audit quality on earnings management and cost of equity capital: evidence from China. Contemp. Account. Res. 28, 892–925. doi: 10.1111/j.1911-3846.2011.01088.x

[ref14] Chen J. Dong W. Tong J. Y. Zhang F. (2018). Corporate philanthropy and investment efficiency: empirical evidence from China. Pac. Basin Finance J. 51, 392–409. doi: 10.1016/j.pacfin.2018.08.008

[ref15] Chen T. Xie L. Zhang Y. (2017). How does Analysts' forecast quality relate to corporate investment efficiency? J. Corp. Finan. 43, 217–240. doi: 10.1016/j.jcorpfin.2016.12.010

[ref16] Chen S. Zheng S. Song T. Wu D. (2011). Government intervention and investment efficiency: evidence from China. J. Corp. Finance 17, 259–271. doi: 10.1016/j.jcorpfin.2010.08.004

[ref17] Cheng B. Ioannou I. Serafeim G. (2014). Corporate social responsibility and access to finance. J. Strat. Manage. 35, 1–23. doi: 10.1002/smj.2131

[ref18] Cheong C. S. Zurbruegg R. (2016). Analyst forecasts and stock price informativeness: some international evidence on the role of audit quality. J. Contemp. Account. Econ. 12, 257–273. doi: 10.1016/j.jcae.2016.09.002

[ref19] Copley P. A. Douthett E. B. (2002). The association between auditor choice, ownership retained, and earnings disclosure by firms making initial public offerings. Contemp. Account. Res. 19, 49–76. doi: 10.1506/U1Y4-CCXT-BPVE-QH58

[ref20] Cui J. Jo H. Na H. (2018). Does corporate social responsibility affect information asymmetry? J. Bus. Ethics 148, 549–572. doi: 10.1007/s10551-015-3003-8

[ref21] Dechow P. M. Sloan R. G. Hutton A. P. (1995). Detecting earnings management. Account. Rev. 70, 193–225.

[ref22] El Ghoul S. Guedhami O. Kwok C. C. Y. Mishra D. R. (2011). Does corporate social responsibility affect the cost of capital? J. Bank. Finance 35, 2388–2406. doi: 10.1016/j.jbankfin.2011.02.007

[ref23] Elaoud A. Jarboui A. (2017). Auditor specialization, accounting information quality and investment efficiency. Res. Int. Bus. Finance 42, 616–629. doi: 10.1016/j.ribaf.2017.07.006

[ref24] Fan J. P. H. Wong T. J. (2005). Do external auditors perform a corporate governance role in emerging markets? Evidence from East Asia. J. Account. Res. 43, 35–72. doi: 10.1111/j.1475-679x.2004.00162.x

[ref25] Francis J. R. Wang D. (2008). The joint effect of investor protection and Big4 audits on earnings quality around the world. Contemp. Account. Res. 25, 157–191. doi: 10.1506/car.25.1.6

[ref26] Francis J. R. Yu M. D. (2009). Big 4 office size and audit quality. Account. Rev. 84, 1521–1552. doi: 10.2308/accr.2009.84.5.1521

[ref27] Goss A. Roberts G. S. (2011). The impact of corporate social responsibility on the cost of Bank loans. J. Bank. Finance 35, 1794–1810. doi: 10.1016/j.jbankfin.2010.12.002

[ref28] Hackenbrack K. E. Hogan C. E. (2002). Market response to earnings surprises conditional on reasons for an auditor change. Contemp. Account. Res. 19, 195–223. doi: 10.1506/5XW7-9CY6-LLJY-BA2F

[ref29] Hai M. Fang Z. Li Z. (2022). Does business Group's conscious of social responsibility enhance its investment efficiency? Evidence from ESG disclosure of China's listed companies. Sustainability 14:4817. doi: 10.3390/su14084817

[ref30] Iatridis G. E. (2011). Accounting disclosures, accounting quality and conditional and unconditional conservatism. Int. Rev. Finance Anal. 20, 88–102. doi: 10.1016/j.irfa.2011.02.013

[ref31] Jones F. L. Raghunandan K. (1998). Client risk and recent changes in the market for audit services. J. Account. Public Policy 17, 169–181. doi: 10.1016/S0278-4254(97)10002-3

[ref32] Khurana I. K. Raman K. K. (2004). Litigation risk and the financial reporting credibility of big 4 versus non-big 4 audits: evidence from Anglo-American countries. Account. Rev. 79, 473–495. doi: 10.2308/accr.2004.79.2.473

[ref33] Kim Y. Park M. S. Wier B. (2012). Is earnings quality associated with corporate social responsibility? Account. Rev. 87, 761–796. doi: 10.2308/accr-10209

[ref34] Kim J. B. Song B. Y. (2011). Auditor quality and loan syndicate structure. Audit. J. Prac. Theory 30, 71–99. doi: 10.2308/ajpt-10144

[ref35] Kolk A. Margineantu A. (2009). Globalization regionalization of accounting firms and their sustainability services. Int. Mark. Rev. 26, 396–410. doi: 10.1108/02651330910971959

[ref36] Lambert R. Leuz C. Verrecchia R. E. (2007). Accounting information, disclosure, and the cost of capital. J. Account. Res. 45, 385–420. doi: 10.1111/j.1475-679X.2007.00238.x

[ref37] Lee J. Kim E. (2020). The influence of corporate environmental responsibility on over Investment behavior: evidence from South Korea. Sustainability 12:1901. doi: 10.3390/su12051901

[ref38] Li X. T. (2009). Managerial entrenchment with strategic information technology: a dynamic perspective. J. Manage. Inf. Syst. 25, 183–204. doi: 10.2753/MIS0742-1222250406

[ref39] Lins K. V. Servaes H. Tamayo A. (2017). Social capital, trust, and firm performance: the value of corporate social responsibility during the financial crisis. J. Finance 72, 1785–1824. doi: 10.1111/jofi.12505

[ref40] Liu Z. Li W. Hao C. Liu H. (2021). Corporate environmental performance and financing constraints: An empirical study in the Chinese context. Corp. Soc. Responsib. Environ. Manage. 28, 616–629. doi: 10.1002/csr.2073

[ref41] Malik M. (2015). Value-enhancing capabilities of CSR: a brief review of contemporary literature. J. Bus. Ethics 127, 419–438. doi: 10.1007/s10551-014-2051-9

[ref42] Mansi S. A. Maxwell W. F. Miller D. P. (2004). Does auditor quality and tenure matter to investors? Evidence from the bond market. J. Account. Res. 42, 755–793. doi: 10.1111/j.1475-679X.2004.00156.x

[ref43] Matten D. Moon J. (2008). “Implicit” and “explicit” CSR: a conceptual framework for a comparative understanding of corporate social responsibility. Acad. Manage. Rev. 33, 404–424. doi: 10.5465/amr.2008.31193458

[ref44] Minutolo M. C. Kristjanpoller W. D. Stakeley J. (2019). Exploring environmental, social, and governance disclosure effects on the S&P 500 financial performance. Bus. Strategy Environ. 28, 1083–1095. doi: 10.1002/bse.2303

[ref45] Mitton T. (2002). A cross-firm analysis of the impact of corporate governance on the east Asian financial crisis. J. Finance Econ. 64, 215–241. doi: 10.1016/S0304-405X(02)00076-4

[ref46] Palazuelos E. Crespo A. H. del Corte J. M. (2018). Accounting information quality and trust as determinants of credit granting to SMEs: the role of external audit. Small Bus. Econ. 51, 861–877. doi: 10.1007/s11187-017-9966-3

[ref47] Pinkowitz L. Stulz R. Williamson R. (2006). Does the contribution of corporate cash holdings and dividends to firm value depend on governance? Across-country analysis. J. Finance 61, 2725–2751. doi: 10.1111/j.1540-6261.2006.01003.x

[ref48] Qin D. Song H. (2009). Sources of investment inefficiency: the case of fixed-asset Investment in China. J. Dev. Econ. 90, 94–105. doi: 10.1016/j.jdeveco.2008.06.001

[ref49] Qureshi M. A. Kirkerud S. Theresa K. Ahsan T. (2019). The impact of sustainability (environmental, social, and governance) disclosure and board diversity on firm value: the moderating role of industry sensitivity. Bus. Strategy Environ. 29, 1199–1214. doi: 10.1002/bse.2427

[ref50] Richardson S. (2006). Over-investment of free cash flow. Rev. Acc. Stud. 11, 159–189. doi: 10.1007/s11142-006-9012-1

[ref51] Samet M. Jarboui A. (2017). How does corporate social responsibility contribute to investment efficiency? J. Multinatl. Finance Manage. 40, 33–46. doi: 10.1016/j.mulfin.2017.05.007

[ref52] Spence M. (1973). Job Market Signaling. Q. J. Econ. 87, 355–374. doi: 10.2307/1882010

[ref53] Stulz R. M. (1990). Managerial discretion and optimal financing policies. J. Finance Econ. 26, 3–27. doi: 10.1016/0304-405X(90)90011-N

[ref54] Sun W. Li X. Geng Y. Yang J. Zhang Y. (2020). Board interlocks and the diffusion of CSR reporting practices: the role of market development. Corp. Soc. Responsibility Environ. Manage 27, 1333–1343. doi: 10.1002/csr.1887

[ref55] Sun W. Zhao C. Cho C. H. (2019). Institutional transitions and the role of financial performance in CSR reporting. Corp. Soc. Responsibility Environ. Manage. 26, 367–376. doi: 10.1002/csr.1688

[ref56] Taliento M. Favino C. Netti A. (2019). Impact of environmental, social, and governance information on economic performance: evidence of a corporate ‘sustainability advantage’ from Europe. Sustainability 11:1738. doi: 10.3390/su11061738

[ref57] Teoh S. H. Wong T. J. (1993). Perceived auditor quality and the earnings response coefficient. Account. Rev. 68, 346–366.

[ref58] Xu P. Bai G. (2019). Board governance, sustainable innovation capability and corporate expansion: empirical data from private listed companies in China. Sustainability 11:3529. doi: 10.3390/su11133529

[ref59] Yoon B. Lee J. Byun R. (2018). Does ESG performance enhance firm value? Evidence from Korea. Sustainability 10:3635. doi: 10.3390/su10103635

[ref60] Zeng S. Qin Y. Zeng G. (2019). Impact of corporate environmental responsibility on investment efficiency: the moderating roles of the institutional environment and consumer environmental awareness. Sustainabilityss 11:4512. doi: 10.3390/su11174512

[ref61] Zhang Q. Loh L. Wu W. (2020). How do environmental, social and governance initiatives affect innovative performance for corporate sustainability? Sustainability 12:3380. doi: 10.3390/su12083380

